# Lower rate of acceptable knee function in adolescents compared with young adults five years after acl reconstruction: results from the swedish national knee ligament register

**DOI:** 10.1186/s12891-022-05727-6

**Published:** 2022-08-19

**Authors:** Baldur Thorolfsson, Michelle Lundgren, Thorkell Snaebjornsson, Jon Karlsson, Kristian Samuelsson, Eric Hamrin Senorski

**Affiliations:** 1 Department of Orthopedics, Sahlgrenska University Hospital, 43180 Gothenburg, Mölndal Sweden; 2 Department of Orthopedics, Institute of Clinical Sciences, Sahlgrenska Academy, University of Gothenburg, Gothenburg, Sweden; 3 Sahlgrenska Sports Medicine Center, Gothenburg, Sweden; 4 Department of Health and Rehabilitation, Institute of Neuroscience and Physiology, Sahlgrenska Academy, University of Gothenburg, Gothenburg, Sweden; 5 Sportrehab Sports Medicine Clinic, Gothenburg, Sweden

**Keywords:** Anterior Cruciate Ligament, ACL, Paediatric, Adolescent, Register

## Abstract

**Background:**

The number of studies with a large cohort of patients that primarily focus on patient-reported outcomes after ACL reconstruction in children and adolescents is limited. The purpose of the present study was to determine whether patient age affects the proportion of patients that achieve a patient-acceptable symptom state (PASS) on the Knee injury and Osteoarthritis Outcome Score (KOOS) subscales one, two, five and 10 years after an ACL reconstruction.

**Methods:**

The patient data in the present study were extracted from the Swedish National Knee Ligament Register (SNKLR). Patients aged between five and 35 years that underwent a primary ACL reconstruction between 1 January 2005 and 31 December 2017 and had completed the KOOS questionnaire at the one-, two-, five- or 10-year follow-up were included. A total of 2,848 patients met the inclusion criteria and were included in the study; 47 paediatric patients (females 5–13, males 5–15 years), 522 adolescents (females 14–19, males 16–19 years) and 2,279 young adults (females 20–35, males 20–35 years). The results from the KOOS were presented as the mean and 95% confidence interval (CI) for the mean. For comparisons between groups, the chi-square test was used for non-ordered categorical variables. For pairwise comparisons between groups, Fisher’s exact test (2-sided) was used for dichotomous variables. All the statistical analyses was set at 5%.

**Results:**

Adolescents reported a significantly lower score than young adults on the KOOS4 at the two- (68.4 vs. 72.1; *P* < 0.05), five- (69.8 vs. 76.0; *P* < 0.05) and 10-year follow-ups (69.8 vs. 78.2; *P* < 0.05). Moreover, a significantly smaller proportion of adolescents achieved a PASS on each of the KOOS subscales when compared with young adults at the five-year follow-up (Symptoms: 83.3% vs. 91.6%; Pain: 42.9% vs. 55.3%; Function in daily living: 31.4% vs. 41.1%; Function in sports and recreational activities: 42.3% vs. 55.7%; Knee-related quality of life: 50.0% vs. 65.0%; *P* < 0.05).

**Conclusions:**

A significantly smaller proportion of adolescents achieved a PASS on each of the KOOS subscales when compared with young adults five years after ACL reconstruction. The results of the present study provide important information for physicians and physiotherapists treating young patients after an ACL injury and they can aid in providing realistic expectations in terms of the mid- and long-term outcomes.

**Level of evidence:**

Prospective Observational Register/Cohort Study, Level II.

**Supplementary Information:**

The online version contains supplementary material available at 10.1186/s12891-022-05727-6.

## Background

Current literature reports better results, in terms of patient-reported outcomes and risk of ACL revision, after anterior cruciate ligament (ACL) reconstruction in adults [1–4] compared with ACL reconstruction in children and adolescents [5–9]. Outcomes in youth ACL literature vary widely [10] and there are very few, if any, studies published with a large cohort of patients that primarily focus on patient-reported outcomes after ACL reconstruction in children and adolescents.

Patient-reported knee complaints vary with age and patient gender in the adult population [11–14] and healthy adolescents and young adults are known to report good to excellent knee function when answering questionnaires such as the Knee injury and Osteoarthritis Outcome Score (KOOS) [12, 14, 15]. In the paediatric and adolescent population, an ACL tear and the following surgical reconstruction is often the largest physical trauma these young patients have encountered. These patients also tend to place high demands on their knees and are eager to return to sport, which may lead to the assumption that they may have difficulties in accepting their knee function post-operatively. However, studies in the adult population have shown acceptable self-reported knee function after ACL reconstruction among the youngest individuals [16, 17].

In the present study, the KOOS was used to assess knee function and outcomes in children and adolescents who underwent ACL reconstruction. The aim of the study was to determine whether patient age, at the time of the ACL injury and reconstruction, affects knee function, reflected by the KOOS, post-operatively at one-, two-, five- and 10-year follow-ups. The aim was also to determine whether patient age, at the time of ACL injury and reconstruction, affects the proportion of patients that achieve a patient-acceptable symptom state (PASS) on the KOOS one, two, five and 10 years after ACL reconstruction. The hypothesis was that adult patients who suffer an ACL rupture after they reach skeletal maturity report higher scores on the KOOS questionnaire post-operatively and achieve a PASS to a greater extent compared with children and adolescents who suffer ACL tears when they are skeletally immature.

## Methods

### The swedish national knee ligament register

The patient data in the present study were extracted from the Swedish National Knee Ligament Register (SNKLR). The register is a nationwide database that uses a web-based protocol for data registration. The register protocol consists of two parts, one is surgeon reported and one is patient reported. The surgeon registers all the surgical procedures performed on the injured knee, including meniscal surgery and the treatment of chondral lesions. The graft type, fixation techniques, patient activity when the ACL injury occurred, time from injury to reconstruction and other concomitant injuries are also reported by the surgeon. The patients register general information about their lifestyle, as well as filling in the KOOS. Recent database validation showed good data quality with more than 97% accuracy when surgeon- and patient-reported data were compared with data from patient journals [18]. As of 2019, the register has been used by more than 90% of all the orthopaedic clinics in Sweden and is publicly financed [18].

### Outcome

Patients register the knee-specific questionnaire, the KOOS, as a part of the patient-reported section in the SNKLR pre-operatively and one, two, five and 10 years post-operatively. The KOOS includes five separately scored sub-scales: Symptoms, Pain, Function in daily living (ADL), Function in sports and recreational activities (sport/rec) and Knee-related quality of life (QoL) [19, 20]. Each subscale on the KOOS ranges from 0 to 100, with 0 indicating extreme symptoms and 100 indicating no symptoms. Moreover, the KOOS4 is an average score for four of the five KOOS subscale scores that is often used when evaluating young patients after ACL reconstruction, as difficulties in ADL tend to be very small if at all present and ceiling effect might therefore be present [19].

Thresholds for a PASS on the KOOS questionnaire have previously been defined for patients after an ACL reconstruction by Muller et al. [21]. In that study, patients were asked to complete the KOOS questionnaire post-operatively, as well as answering the question “Taking account of all the activity you have during your daily life, your level of pain and also your activity limitations and participation restrictions, do you consider the current state of your knee satisfactory? (Yes or No)”. The PASS threshold (sensitivity, specificity) was 57.1 (0.78, 0.67) for the KOOS symptoms, 88.9 (0.82, 0.81) for the KOOS pain, 100.0 (0.70, 0.89) for the KOOS ADL, 75.0 (0.87, 0.88) for the KOOS sport/rec and 62.5 (0.82, 0.85) for the KOOS QoL. The same thresholds were used to determine acceptable knee function in the present study.

### Patients

**Fig. 1:**
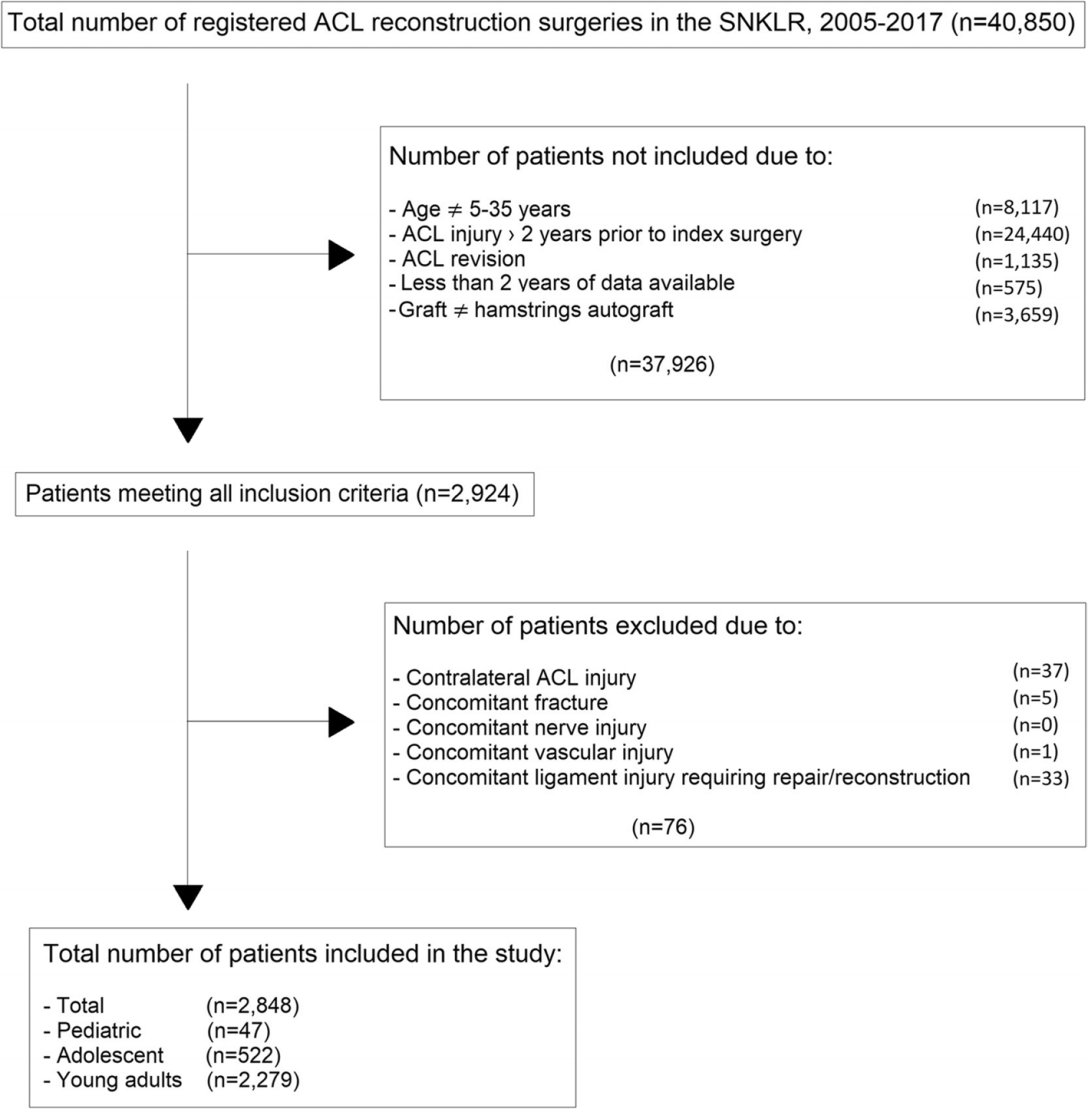
Flow chart of inclusion and exclusion criteria

Patients aged between five and 35 years who underwent a primary ACL reconstruction between 1 January 2005 and 31 December 2017 and had completed the KOOS questionnaire at the one, two-, five- or 10-year follow-up were eligible for inclusion. Patients were excluded from the study if they underwent surgery with a graft other than a hamstring autograft, had a concomitant nerve injury, vascular injury, fracture, grade III injury to the medial collateral ligament (MCL) or the lateral collateral ligament (LCL), an injury to the posterior cruciate ligament (PCL) or if they were operated on more than two years after the ACL injury had occurred. A flow chart of the inclusion and exclusion criteria can be seen in Fig. 1.

The cohort was stratified into age groups of females aged 5–13, 14–19 and 20–35 years and males aged 5–15, 16–19 and 20–35 years, as seen in Table 1. This was done to include one group of skeletally immature individuals with open physes, a second group of individuals who underwent ACL reconstruction at, or just after the time of, physeal closure and a third reference group of skeletally mature young adults. Radiographs are needed to thoroughly determine skeletal age and maturity on an individual basis, but, to generalise the cohort, the age of skeletal maturity was set at 14 years in females and 16 years in males, as this is generally regarded as a fair estimation [22–24].

**Table 1:** Definition of age groups in the study

	**Female**	**Male**
Children	5–13 years*	5–15 years*
Adolescents	14–19 years*	16–19 years*
Young adults	20–35 years	20–35 years

^*^To generalise the cohort, the age of skeletal maturity was set at 14 years in females and 16 years in males

According to Swedish law (2008:355), written consent need not be obtained for national registers of this kind in Sweden and participation is voluntary for both patients and surgeons. Patients are presented with information on the SNKLR and are free to withdraw from participation at any time. The extracted data are anonymous and patient age and gender are only identifiable to authorised personnel from the patient´s social security number. All the methods were performed in accordance with the Declaration of Helsinki and the study was approved by the regional Ethical Review Board in Stockholm, Sweden (review ref: 2011/337–31/3).

### Variables and outcome

The following data were extracted from the SNKLR; patient age, weight, patient gender, concomitant injuries registered at ACL reconstruction, graft type, activity when the injury occurred and KOOS measured pre-operatively and at one, two, five and 10 years post-operatively. Cross-sectional cohorts were utilised at each follow-up to maximise the number of patients. Follow-up started at index surgery and finished on 31 December 2017. The primary study outcome was achieving a PASS on each subscale of the KOOS.

### Statistical analysis

Statistical analysis was performed with the SAS statistical analysis system (SAS/STAT, v 14.2; SAS Institute Inc., Cary, NC, USA). For categorical variables, count (n) and proportion (%) were presented. For continuous variables, the mean and standard deviations (SD) and the median with minimum to maximum together with the n of patients were presented. The results from the KOOS were presented as the mean and 95% confidence interval (CI) for the mean. For comparisons between groups, the chi-square test was used for non-ordered categorical variables. For pairwise comparisons between groups, Fisher’s exact test (2-sided) was used for dichotomous variables and Fisher´s non parametric permutation test was used for continuous variables. The significance level in all the statistical analyses was set at 5%.

## Results

During the study period, a total of 40,850 ACL reconstructions were registered in the SNKLR. Of these, 2,848 patients met the inclusion criteria and were included in the study: 47 paediatric patients (mean age 13.6 ± 1.6 years), 522 adolescents (mean age 17.4 ± 1.4 years) and 2,279 young adults (mean age 27.0 ± 4.5 years). For all age groups, pivoting sports, such as basketball, football, team handball and floorball, were the most common cause of ACL injury. Associated injuries to the joint cartilage were more common in the older age groups (P < 0.05). However, associated injuries to the lateral meniscus, medial meniscus, LCL and MCL did not differ significantly between the groups. The demographic characteristics of the study groups are presented in Table 2. Demographic data of the study groups at each follow-up is presented as appendix tables.

**Table 2:** Demographic data of the study groups

	**Total** **(*n* = 2,848)**	**Paediatric** **(*n* = 47)**	**Adolescent** **(*n* = 522)**	**Young adult** **(*n* = 2,279)**
**Gender**				
Male	1,699 (59.7%)	34 (72.3%)	208 (39.8%)	1,457 (63.9%)
Female	1,149 (40.3%)	13 (27.7%)	314 (60.2%)	822 (36.1%)
**Age at index surgery**	25.0 (5.7) 25 (9; 35)	13.6 (1.6) 14 (9; 15)	17.4 (1.4) 18 (14; 19)	27.0 (4.5) 26 (20; 35)
**Activity at ACL injury**				
Pivoting sports	1,759 (61.8%)	23 (48.9%)	355 (68.0%)	1,381 (60.6%)
Non-pivoting sports	64 (2.2%)	1 (2.1%)	12 (2.3%)	51 (2.2%)
Martial arts	75 (2.6%)	1 (2.1%)	8 (1.5%)	66 (2.9%)
Winter sports	382 (13.4%)	8 (17.0%)	62 (11.9%)	312 (13.7%)
Other	560 (19.7%)	14 (29.8%)	85 (16.3%)	461 (20.2%)
Missing	8 (0.3%)	0	0	8 (0.4%)
**Groups of femoral fixation**				
Cortical suspensory fixation	1,234 (43.3%)	32 (68.1%)	209 (40.0%)	993 (43.6%)
Adjustable cortical suspensory fixation	416 (14.6%)	11 (23.4%)	73 (14.0%)	332 (14.6%)
Screw fixation	340 (11.9%)	1 (2.1%)	72 (13.8%)	267 (11.7%)
Intratunnel transfixation	828 (29.1%)	3 (6.4%)	165 (31.6%)	660 (29.0%)
Other	17 (0.6%)	0	3 (0.6%)	14 (0.6%)
Femur fixation missing	13 (0.5%)	0	0	13 (0.6%)
**Groups of tibial fixation**				
Cortical suspensory fixation	22 (0.8%)	0	3 (0.6%)	19 (0.8%)
Adjustable cortical suspensory fixation	147 (5.2%)	4 (8.5%)	32 (6.1%)	111 (4.9%)
Screw fixation	1,981 (69.6%)	36 (76.6%)	360 (69.0%)	1,585 (69.5%)
Bioabsorbable screw	544 (19.1%)	4 (8.5%)	101 (19.3%)	439 (19.3%)
Intratunnel transfixation	82 (2.9%)	0	17 (3.3%)	65 (2.9%)
Other	52 (1.8%)	3 (6.4%)	8 (1.5%)	41 (1.8%)
Tibial fixation missing	20 (0.7%)	0	1 (0.2%)	19 (0.8%)
**Concomitant injuries**				
Medial meniscus	1,063 (37.3%)	13 (27.7%)	185 (35.4%)	865 (38.0%)
Lateral meniscus	690 (24.2%)	14 (29.8%)	143 (27.4%)	533 (23.4%)
Cartilage injury	956 (33.6%)	5 (10.6%)	126 (24.1%)	825 (36.2%)
MCL	18 (0.6%)	0	1 (0.2%)	17 (0.7%)
LCL	6 (0.2%)	0	1 (0.2%)	5 (0.2%)

*ACL* Anterior cruciate ligament, *LCL* Lateral collateral ligament, *MCL* Medial collateral ligament

### Knee function

**Fig. 2:**
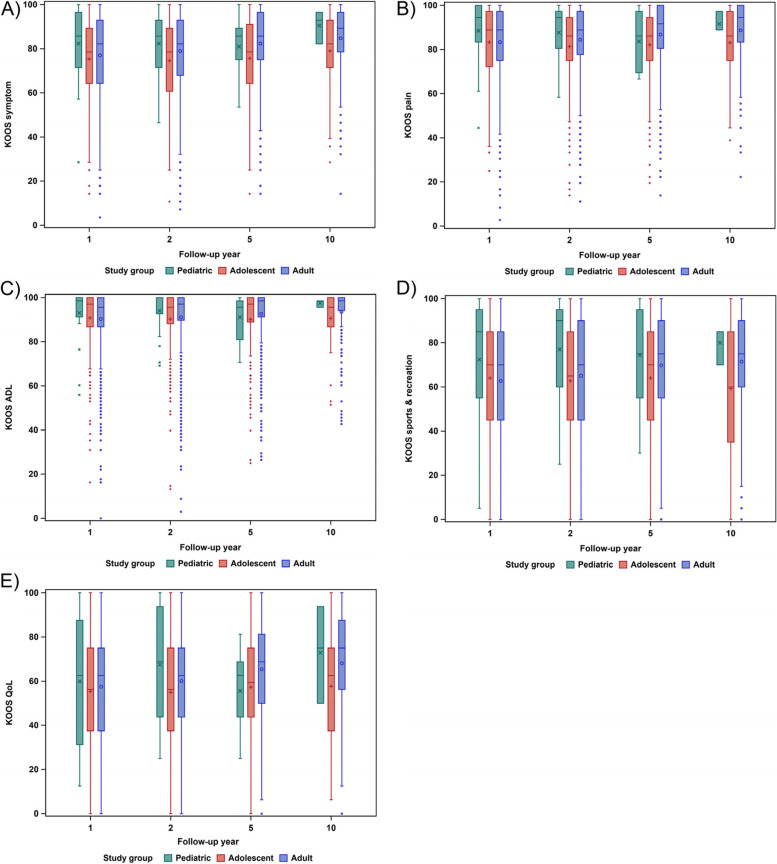
Box plots displaying the interquartile range, median and mean of the Knee injury and Osteoarthritis Outcome Score (KOOS) subscales over time. One-year total number of patients (*N*) = 1,366; 27 children, 275 adolescents, 1,064 young adults. Two-year *N* = 1,211; 25 children, 234 adolescents, 952 young adults. Five-year *N* = 822; nine children, 156 adolescents, 657 young adults. Ten-year *N* = 260; three children, 47 adolescents, 210 young adults

Of the 2,848 patients included in the study, a total of 1,366, 27 children, 275 adolescents and 1,064 young adults, answered the KOOS questionnaire at the one-year follow-up. A total of 1,211 patients, 25 children, 234 adolescents and 952 young adults, answered the KOOS questionnaire at the two-year follow-up. A total of 822 patients, nine children, 156 adolescents and 657 young adults, answered the KOOS questionnaire at the five-year follow-up. A total of 260 patients, three children, 47 adolescents and 210 young adults, answered the KOOS questionnaire at the 10-year follow-up. Adolescents reported significantly lower scores than young adults on the KOOS4 at the two-, five- and 10-year follow-ups (*P* < 0.05). The results from the KOOS questionnaire are shown in Fig. 2 and Table 3.

**Table 3:** Results of the Knee injury and Osteoarthritis Outcome Score (KOOS) subscales at each visit showing means, standard deviations, medians and interquartile ranges as well as number of patients at each visit

					**Test between groups** ***p*-value**		
	**Total**	**Paediatric**	**Adolescent**	**Adult**	**Paediatric vs Adolescent**	**Paediatric vs Adult**	**Adolescent vs Adult**
**- 1 year -**							
**KOOS—Pain**	83.4 (16.6) 88.9 (2.8; 100) (82.5; 84.3) *n* = 1365	88.5 (14.5) 94.4 (44.4; 100) (82.7; 93.5) *n* = 27	83.3 (16.7) 88.9 (25; 100) (81.2; 85.2) *n* = 275	83.3 (16.6) 88.9 (2.8; 100) (82.3; 84.3) *n* = 1063	0.10	0.091	0.98
**KOOS—Symtom**	76.8 (18.4) 82.1 (3.6; 100) (75.8; 77.7) *n* = 1365	82.3 (16.9) 85.7 (28.6; 100) (75.7; 88.2) *n* = 27	75.3 (18.6) 78.6 (14.3; 100) (73.1; 77.5) *n* = 275	77.0 (18.3) 82.1 (3.6; 100) (75.9; 78.1) *n* = 1063	0.054	0.13	0.18
**KOOS—ADL**	90.4 (14.1) 95.6 (0; 100) (89.7; 91.2) *n* = 1365	93.0 (11.5) 98.5 (55.9; 100) (88.3; 96.8) *n* = 27	90.8 (14.1) 97.1 (16.2; 100) (89.1; 92.4) *n* = 274	90.3 (14.2) 95.6 (0; 100) (89.4; 91.1) *n* = 1064	0.45	0.32	0.63
**KOOS – Sports & recreation**	63.2 (27.7) 70 (0; 100) (61.7; 64.6) *n* = 1365	72.4 (29.6) 85 (5; 100) (60.7; 82.7) *n* = 27	64.1 (27.7) 70 (0; 100) (60.8; 67.3) *n* = 274	62.7 (27.7) 70 (0; 100) (61.1; 64.4) *n* = 1064	0.14	0.070	0.48
**KOOS – QoL**	57.1 (24.1) 56.3 (0; 100) (55.8; 58.4) *n* = 1366	60.0 (29.7) 62.5 (12.5; 100) (48.9; 71.0) *n* = 27	55.4 (24.0) 56.3 (0; 100) (52.6; 58.2) *n* = 275	57.5 (24.0) 62.5 (0; 100) (56.1; 58.9) *n* = 1064	0.37	0.63	0.20
**KOOS4**	70.1 (19.4) 73.5 (9.1; 100) (69.0; 71.1) *n* = 1366	75.8 (19.9) 80.3 (22.6; 100) (68.0; 82.8) *n* = 27	69.5 (19.4) 72.2 (10.7; 100) (67.1; 71.7) *n* = 275	70.1 (19.4) 73.7 (9.1; 100) (68.9; 71.2) *n* = 1064	0.099	0.13	0.63
**- 2 years -**							
**KOOS—Pain**	83.9 (16.9) 88.9 (11.1; 100) (82.9; 84.8) *n* = 1211	87.7 (13.2) 94.4 (58.3; 100) (82.4; 92.4) *n* = 25	81.4 (17.8) 86.1 (13.9; 100) (79.0; 83.6) *n* = 234	84.4 (16.7) 88.9 (11.1; 100) (83.3; 85.4) *n* = 952	0.067	0.34	0.017
**KOOS—Symtom**	78.2 (18.1) 82.1 (7.1; 100) (77.2; 79.2) *n* = 1211	82.3 (16.5) 85.7 (46.4; 100) (75.7; 88.4) *n* = 25	74.6 (18.5) 78.6 (10.7; 100) (72.1; 77.0) *n* = 234	78.9 (18.0) 82.1 (7.1; 100) (77.7; 80.1) *n* = 952	0.038	0.37	0.0014
**KOOS—ADL**	90.9 (14.2) 97.1 (2.9; 100) (90.1; 91.7) *n* = 1211	93.9 (9.4) 100 (69.1; 100) (90.0; 97.3) *n* = 25	90.2 (14.6) 95.6 (13.2; 100) (88.3; 92.0) *n* = 234	91.0 (14.2) 97.1 (2.9; 100) (90.1; 91.9) *n* = 952	0.20	0.31	0.42
**KOOS – Sports & recreation**	64.8 (27.5) 70 (0; 100) (63.3; 66.4) *n* = 1210	77.0 (24.5) 90 (25; 100) (67.1; 85.9) *n* = 25	62.8 (27.7) 65 (0; 100) (59.2; 66.3) *n* = 234	65.0 (27.5) 70 (0; 100) (63.3; 66.8) *n* = 951	0.011	0.026	0.27
**KOOS – QoL**	59.2 (23.9) 62.5 (0; 100) (57.9; 60.6) *n* = 1211	67.5 (26.3) 68.8 (25; 100) (57.3; 77.5) *n* = 25	55.0 (23.9) 56.3 (0; 100) (52.0; 58.0) *n* = 234	60.1 (23.7) 62.5 (0; 100) (58.6; 61.6) *n* = 952	0.016	0.13	0.0034
**KOOS4**	71.5 (19.7) 75.2 (6.2; 100) (70.4; 72.6) *n* = 1211	78.6 (18.2) 80.7 (43.7; 100) (71.4; 85.4) *n* = 25	68.4 (19.6) 69.3 (6.2; 100) (65.9; 70.9) *n* = 234	72.1 (19.7) 75.9 (8.6; 100) (70.8; 73.3) *n* = 952	0.0098	0.094	0.012
**- 5 years -**							
**KOOS—Pain**	85.9 (16.4) 91.7 (13.9; 100) (84.8; 87.0) *n* = 822	83.6 (14.2) 86.1 (66.7; 100) (74.1; 92.9) *n* = 9	82.1 (18.3) 86.1 (19.4; 100) (79.1; 84.9) *n* = 156	86.8 (15.8) 91.7 (13.9; 100) (85.6; 88.0) *n* = 657	0.87	0.52	0.0018
**KOOS—Symtom**	81.0 (17.6) 85.7 (14.3; 100) (79.8; 82.2) *n* = 822	81.0 (14.9) 85.7 (53.6; 100) (70.8; 90.1) *n* = 9	75.7 (19.4) 78.6 (14.3; 100) (72.6; 78.7) *n* = 156	82.3 (16.9) 85.7 (14.3; 100) (81.0; 83.6) *n* = 657	0.45	0.77	0.0002
**KOOS—ADL**	92.1 (13.4) 98.5 (25; 100) (91.2; 93.0) *n* = 822	91.0 (10.8) 95.6 (70.6; 100) (83.5; 97.6) *n* = 9	90.1 (15.5) 97.1 (25; 100) (87.6; 92.5) *n* = 156	92.6 (12.8) 98.5 (26.5; 100) (91.6; 93.6) *n* = 657	0.96	0.64	0.046
**KOOS – Sports & recreation**	68.7 (26.4) 75 (0; 100) (66.9; 70.5) *n* = 822	74.4 (24.8) 75 (30; 100) (57.1; 89.5) *n* = 9	64.1 (27.7) 70 (0; 100) (59.7; 68.4) *n* = 156	69.7 (26.0) 75 (0; 100) (67.7; 71.7) *n* = 657	0.28	0.65	0.020
**KOOS – QoL**	63.7 (22.9) 68.8 (0; 100) (62.1; 65.2) *n* = 822	55.6 (20.6) 62.5 (25; 81.3) (42.0; 68.8) *n* = 9	57.3 (23.2) 59.4 (0; 100) (53.7; 60.9) *n* = 156	65.3 (22.6) 68.8 (0; 100) (63.5; 67.1) *n* = 657	0.86	0.22	0.0002
**KOOS4**	74.8 (19.0) 79.1 (11.8; 100) (73.5; 76.1) *n* = 822	73.6 (16.7) 71.2 (52.2; 92.2) (62.6; 84.4) *n* = 9	69.8 (20.2) 72.4 (11.8; 100) (66.6; 72.9) *n* = 156	76.0 (18.6) 80.5 (14.3; 100) (74.6; 77.5) *n* = 657	0.59	0.66	0.0006
**- 10 years -**							
**KOOS—Pain**	87.7 (14.8) 91.7 (22.2; 100) (85.9; 89.4) *n* = 260	91.7 (4.8) 88.9 (88.9; 97.2) (88.9; 97.2) *n* = 3	83.0 (15.4) 86.1 (38.9; 100) (78.5; 87.2) *n* = 47	88.7 (14.5) 94.4 (22.2; 100) (86.7; 90.6) *n* = 210	0.38	0.92	0.025
**KOOS—Symtom**	83.8 (16.1) 89.3 (14.3; 100) (81.8; 85.7) *n* = 260	90.5 (7.4) 92.9 (82.1; 96.4) (82.1; 96.4) *n* = 3	79.0 (17.7) 82.1 (28.6; 100) (73.6; 84.0) *n* = 47	84.7 (15.6) 89.3 (14.3; 100) (82.6; 86.7) *n* = 210	0.28	0.64	0.034
**KOOS—ADL**	93.0 (12.3) 98.5 (42.7; 100) (91.5; 94.5) *n* = 260	97.6 (1.7) 98.5 (95.6; 98.5) (95.6; 98.5) *n* = 3	90.6 (13.6) 95.6 (51.5; 100) (86.4; 94.2) *n* = 47	93.5 (12.0) 98.5 (42.7; 100) (91.8; 95.1) *n* = 210	0.47	0.84	0.15
**KOOS – Sports & recreation**	69.3 (26.7) 75 (0; 100) (66.2; 72.6) *n* = 260	80.0 (8.7) 85 (70; 85) (70.0; 85.0) *n* = 3	59.3 (28.5) 60 (0; 100) (50.8; 67.5) *n* = 47	71.5 (26.0) 75 (0; 100) (67.9; 74.9) *n* = 210	0.24	0.70	0.0058
**KOOS – QoL**	66.3 (23.9) 75 (0; 100) (63.5; 69.1) *n* = 260	72.9 (21.9) 75 (50; 93.8) (50.0; 93.8) n = 3	57.7 (24.8) 62.5 (6.3; 100) (50.6; 64.8) *n* = 47	68.1 (23.4) 75 (0; 100) (65.0; 71.2) *n* = 210	0.36	0.86	0.0085
**KOOS4**	76.8 (18.6) 82.4 (10.4; 100) (74.6; 79.0) *n* = 260	83.8 (10.3) 85.4 (72.8; 93.1) (72.8; 93.1) *n* = 3	69.8 (19.5) 72.6 (26; 98.4) (64.0; 75.3) *n* = 47	78.2 (18.2) 83.9 (10.4; 100) (75.8; 80.6) *n* = 210	0.22	0.70	0.0058

*KOOS* Knee injury and osteoarthritis outcome score, *ADL* Function in daily living, *QoL* Knee-related quality of life

### Patient acceptable symptom state

#### Symptoms

**Fig. 3:**
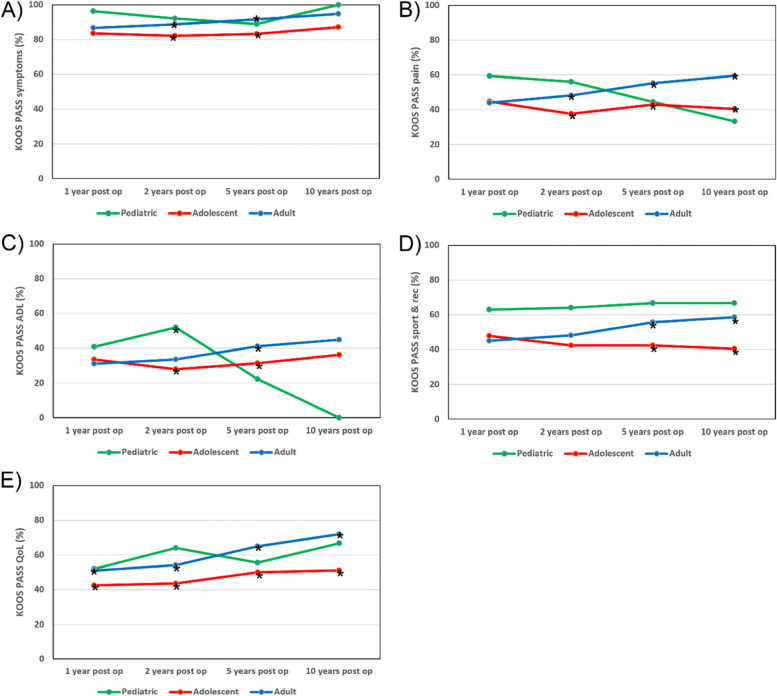
Line charts showing the proportion of patients achieving a patient-acceptable symptom state (PASS) on each of the Knee injury and Osteoarthritis Outcome Score (KOOS) subscales over the 10-year follow-up period. All significant comparisons between groups where *P* < 0.05 are marked with *. One-year total number of patients (*N*) = 1,366; 27 children, 275 adolescents, 1,064 young adults. Two-year *N* = 1,211; 25 children, 234 adolescents, 952 young adults. Five-year *N* = 822; nine children, 156 adolescents, 657 young adults. Ten-year *N* = 260; three children, 47 adolescents, 210 young adults

A significantly smaller proportion of adolescents achieved a PASS than young adults on the Symptoms subscale at two (82.1% vs. 88.8%; *P* < 0.05) and five (83.3% vs. 91.6%; *P* < 0.05) years. However, there was no difference between any of the groups at the one- and 10-year follow-ups and the paediatric age group did not show any difference in terms of the PASS for the Symptoms subscale at any of the follow-ups when compared with adolescents and young adults (Fig. 3).

#### Pain

A significantly smaller proportion of adolescents achieved a PASS than young adults on the Pain subscale at two (37.6% vs. 48.1%%; *P* < 0.05), five (42.9% vs. 55.3%; *P* < 0.05) and 10 (40.4% vs. 59.5%; *P* < 0.05) years. There were no differences between any of the groups at the one-year follow-up and the paediatric age group did not show any differences in terms of the PASS on the Pain subscale at any of the follow-ups when compared with adolescents and young adults (Fig. 3).

#### ADL

A larger proportion of paediatric patients achieved a PASS on the ADL subscale when compared with adolescents (52.0% vs. 27.8%; *P* < 0.05) at the two-year follow-up. A significantly smaller proportion of adolescents achieved a PASS than young adults (31.4% vs. 41.1%; *P* < 0.05) at the five-year follow-up. However, there was no difference between any of the groups in terms of the PASS on the ADL subscale at the one- and 10-year follow-ups (Fig. 3).

#### Sport/recreation

A significantly smaller proportion of adolescents achieved a PASS compared with young adults on the Sport/rec subscale at five (42.3% vs. 55.7%; *P* < 0.05) and 10 (40.4% vs. 58.6%; *P* < 0.05) years. However, there was no difference between adolescents and young adults at the one- and two-year follow-ups. The paediatric age group did not show a statistically significant difference in terms of the PASS on the Sport/rec subscale at any of the follow-ups when compared with adolescents and young adults (Fig. 3).

#### QoL

A significantly smaller proportion of adolescents achieved a PASS than young adults on the QoL subscale at one (42.5% vs. 51.0%; *P* < 0.05), two (43.6% vs. 54.1%; *P* < 0.05), five (50.0% vs. 65.0%; *P* < 0.05) and 10 (51.1% vs. 71.9%; *P* < 0.05) years. However, the paediatric age group did not show any differences in terms of the PASS on the Qol subscale at any of the follow-ups when compared with adolescents and young adults (Fig. 3).

## Discussion

The main finding in this large population-based register study was that a significantly smaller proportion of adolescents achieved a PASS on all the KOOS subscales when compared with young adults at the five-year follow-up. Moreover, a similar pattern was seen at the one-, two- and 10-year follow-ups, although it was not statistically significant for each of the KOOS subscales at every follow-up. The present study reveals that a significant proportion of patients were not satisfied with their current knee state at all follow-ups, on all KOOS subscales up to 10 years after surgery, with the exception of KOOS Symptoms, where > 80% of the patients were satisfied at all follow-ups.

The present study is one of the first to look at the PASS on the KOOS separately between children and adolescents, although Hamrin Senorski et al. [16] have previously reported that young age is a favourable factor that increases the odds of early acceptable knee function. Young adults increased their KOOS at each follow-up on each subscale and a larger proportion of young adults achieved acceptable knee function over time. Adolescents generally reported a PASS to a lesser extent, when compared with the paediatric age group, although the proportion of adolescents reporting acceptable knee function was consistent over the 10-year time period.

This finding highlights the importance of thorough pre-operative planning and shared decision-making with the patient and the patient’s parents before deciding on early surgical reconstruction in children and adolescents and care should be taken not to expect the same post-operative outcome in that age group as in young adults.

Desai et al. [1] have previously reported higher KOOS scores among patients aged 0–19 years on all KOOS subscales at the one- and two-year follow-ups when compared with patients 20–29 years of age. The present study, however, showed that adolescents obtained lower scores than young adults in most categories of the KOOS after one and two years. That difference can probably be explained by the fact that the group of adolescents in the present study did not include paediatric patients who, on the other hand, reported higher KOOS scores at those follow-ups. This suggests a difference in the patient-reported outcomes after an ACL reconstruction between adolescents and paediatric patients, thereby indicating that separating these groups in future studies would be valuable. Moreover, there was a similar trend in the study by Desai et al. [1] and the present study, where the young adults showed a greater increase on the KOOS between the two- and five-year follow-ups when compared with adolescents.

In an American cohort, the MOON Knee Group [25] reported significantly improved KOOS two years post-operatively compared with baseline. That study comprised 1,379 patients with a median age of 24 at the two-year follow-up (17–35). Their finding is partly comparable with the young adults in the present study, indicating that the greatest increase in the KOOS occurs during the first years after ACL reconstruction. However, it differs in that the largest increase in the present study occurred between the two- and five-year follow-ups. From the same cohort, the MOON Knee Group reported that the higher scores achieved on the KOOS were maintained at the six- and 10-year follow-ups. A similar trend was seen in this study, where, on average, higher scores on the KOOS are achieved at five and 10 years post-operatively. The same pattern is seen when examining the proportion of young adults achieving a PASS on the KOOS. This proportion increases after the one-year follow-up and the largest proportion achieving a PASS is seen at the 10-year follow-up.

Samuelsson et al. [17] reported equivalent KOOS scores after ACL reconstruction between the one- and two-year follow-ups. The same thing was seen in the present study, but we noted an interesting increase in the KOOS and in the proportion of PASS among adolescents and young adults from the two-year to the five-year follow-up. We claim that a PASS might be more useful in evaluating the patients’ experience of the outcome. Even though changes in the KOOS itself can show statistically significant changes, this does not necessarily reflect a corresponding clinical improvement. For example, the KOOS in the QoL subcategory is low on average, suggesting that the patients are generally dissatisfied. However, the PASS threshold for the QoL is set at a comparably low KOOS. As a result, a low KOOS for QoL can still represent a condition in which patients regard their symptoms as acceptable. For this reason, the addition of a PASS as a cut-off makes it possible to better interpret the meaning of changes in KOOS scores in the clinical routine [26].

In the present study, a smaller proportion of adolescents achieved a PASS on each of the KOOS subscales when compared with young adults at the five-year follow-up, which is interesting, bearing in mind that associated injuries to the joint cartilage and medial meniscus are more common in the older age groups and highest among the young adults. One possible explanation for the poorer outcome in adolescents when compared with young adults could be that adolescents tend to return to high activity levels and high-impact activities earlier than young adults after an ACL injury [27]. It might also be worth considering whether the associated injuries are significant predictors of the outcome on the KOOS In a short-term follow-up from the Norwegian Knee Ligament Register, LaPrade et al. [28] reported that, at the two-year follow-up, no significant differences were seen on the KOOS between patients with an isolated ACL reconstruction and patients with an ACL reconstruction together with a concomitant lateral meniscus repair, lateral meniscus resection or medial meniscus resection. However, patients with an ACL reconstruction and a medial meniscus repair obtained a significantly lower KOOS score on two of the subscales, Symptoms and QoL, in comparison with those with an isolated ACL reconstruction. The cohort from the MOON Knee Group [25] with a 10-year follow-up reported that lesions on the MCL or LCL, as well as meniscal lesions with treatment at the time of the ACL reconstruction, were not significant risk factors for an inferior 10-year outcome measured on the KOOS. This suggests that age and associated levels of physical activity and sport could be more important than most of these associated injuries at baseline when predicting the post-operative KOOS.

We consider that the large sample size that provides precision and high statistical power in the adolescent and young adult age groups is a strength in the present study. The study is a population-based register study with a large number of patients and this allows the generalisation of the findings, at least nationally. Moreover, the different age grouping of males and females is seen as a strength, as the time of skeletal maturity differs between the sexes and therefore allows the most accurate grouping possible in terms of skeletal maturity.

### Limitations

The main limitation of this study is the small number of paediatric patients, resulting in statistically insignificant results in that age group and probably underpowered analyses. Another limitation is that individual radiographs were not available to determine skeletal maturity. Instead, skeletal maturity was generalised, depending on age, which may have caused some individuals to fall into the wrong category. A further limitation is that different patient cohorts answered the KOOS questionnaire at different follow-ups. Another weakness is that the defined PASS thresholds for the KOOS subscales have only been validated for individuals one to five years after ACL reconstruction [21] and it is therefore doubtful that the same threshold for knee satisfaction applies at the 10-year follow-up. Further, in the study defining the PASS thresholds, Muller et al. [21] included patients between the ages of 14 and 50 at the time of index surgery. As a result, the PASS thresholds may be different for the KOOS extracted from the 10-year follow-up and also for the group of paediatric patients in the present study. We divided the groups based on age in an attempt to mirror skeletal maturity and therefore used different age cut-offs in the paediatric and adolescent age groups based on patient gender. This could of course influence the outcome, as cognitive, emotional and social development may also impact KOOS outcomes and the PASS. The last limitation we would like to mention is that KOOS is validated for patients 13–79 years of age and has been a part of the SNKLR data set from the beginning of the register. However, KOOS child version for children aged 9–12 years has later become available but has not been implemented in the register yet. This somewhat limits KOOS data for paediatric patients under the age of 13.

Future studies should aim to include a larger cohort of paediatric patients, possibly by contacting those individuals more frequently to encourage them to respond and answer the KOOS questionnaire at two-, five- and 10-year follow-ups.

## Conclusions

A significantly smaller proportion of adolescents perceive their knee function as acceptable when compared with young adults five years after ACL reconstruction. Adult patients report better knee function post-operatively compared with children and adolescents. The results of the present study provide important information to physicians and physiotherapists treating young patients after an ACL injury that may aid in providing realistic expectations regarding the long-term outcome. Future studies should separate paediatric patients from adolescents in terms of outcome after an ACL reconstruction.

## Supplementary Information

**Additional file 1:** Appendix **Table 1. **Demographic data of the study groups at the one-year follow-up. **Table 2. **Demographic data of the study groups at the two-year follow-up. **Table 3. **Demographic data of the study groups at the five-year follow-up. **Table 4. **Demographic data of the study groups at the 10-year follow-up.

## Data Availability

The datasets used and/or analysed during the current study are available from the corresponding author in response to a reasonable request.

## Abbreviations

ACL: Anterior cruciate ligament
ADL: Function in daily living
CI: Confidence interval
KOOS: Knee injury and osteoarthritis outcome score
LCL: Lateral collateral ligament
MCL: Medial collateral ligament
N: Count
PASS: Patient-acceptable symptom state
PCL: Posterior cruciate ligament
QoL: Knee-related quality of life
SD: Standard deviation
SNKLR: Swedish national knee ligament register
Sport/rec: Function in sports and recreational activities
