# Supplementary material for: Lower rate of acceptable knee function in adolescents compared with young adults five years after acl reconstruction: results from the swedish national knee ligament register
Source: BMC Musculoskelet Disord. 2022 Aug 19;23:793. doi: 10.1186/s12891-022-05727-6 (PMC9389739; doi:10.1186/s12891-022-05727-6)
Supplement: Supplementary file 1 — Additional file 1: Appendix Table 1. Demographic data of the study groups at the one-year follow-up. Table 2. Demographic data of the study groups at the two-year follow-up. Table 3. Demographic data of the study groups at the five-year follow-up. Table 4. Demographic data of the study groups at the 10-year follow-up. [file 12891_2022_5727_MOESM1_ESM.docx]

**Appendix**

**Table 4.** Demographic data of the study groups at the one-year follow-up

|  | **Total (n=1,365)** | **Paediatric (n=47)** | **Adolescent (n=275)** | **Young adult (n=1,063)** |
| --- | --- | --- | --- | --- |
| **Gender** |  |  |  |  |
| Male | 713 (52.2%) | 19 (70.4%) | 93 (33.8%) | 601 (56.5%) |
| Female | 652 (47.8%) | 8 (29.6%) | 182 (66.2%) | 462 (43.5%) |
| **Age at index surgery** | 24.8 (5.8) 25 (9; 35) | 13.7 (1.7) 15 (9; 15) | 17.3 (1.4) 17 (14; 19) | 27.0 (4.6) 27 (20; 35) |
| **Activity at ACL injury** |  |  |  |  |
| Pivoting sports | 445 (62.9%) | 6 (31.6%) | 65 (69.9%) | 374 (62.8%) |
| Non-pivoting sports | 12 (1.7%) | 1 (5.3%) | 1 (1.1%) | 10 (1.7%) |
| Martial arts | 23 (3.2%) | 1 (5.3%) | 2 (2.2%) | 20 (3.4%) |
| Winter sports | 68 (9.6%) | 4 (21.1%) | 9 (9.7%) | 55 (9.2%) |
| Other | 160 (22.6%) | 7 (36.8%) | 16 (17.2%) | 137 (23.0%) |
| Missing | 5 (0.4%) | 0 | 0 | 5 (0.5%) |
| **Groups of femoral fixation** |  |  |  |  |
| Cortical suspensory fixation | 568 (41.6%) | 16 (59.3%) | 109 (39.6%) | 443 (41.7%) |
| Adjustable cortical suspensory fixation | 203 (14.9%) | 7 (25.9%) | 37 (13.5%) | 159 (15.0%) |
| Screw fixation | 158 (11.6%) | 1 (3.7%) | 33 (12.0%) | 124 (11.7%) |
| Intratunnel transfixation | 425 (31.1%) | 3 (11.1%) | 96 (34.9%) | 326 (30.7%) |
| Other | 4 (0.3%) | 0 | 0 | 4 (0.4%) |
| Femur fixation missing | 7 (0.5%) | 0 | 0 | 7 (0.7%) |
| **Groups of tibial fixation** |  |  |  |  |
| Cortical suspensory fixation | 8 (0.6%) | 0 | 1 (0.4%) | 7 (0.7%) |
| Adjustable cortical suspensory fixation | 65 (4.8%) | 1 (3.7%) | 13 (4.7%) | 51 (4.8%) |
| Screw fixation | 960 (70.3%) | 21 (77.8%) | 194 (70.5%) | 745 (70.1%) |
| Bioabsorbable screw | 249 (18.2%) | 2 (7.4%) | 55 (20.0%) | 192 (18.1%) |
| Intratunnel transfixation | 43 (3.2%) | 0 | 9 (3.3%) | 34 (3.2%) |
| Other | 30 (2.2%) | 3 (11.1%) | 3 (1.1%) | 24 (2.3%) |
| Tibial fixation missing | 10 (0.7%) | 0 | 0 | 10 (0.9%) |
| **Concomitant injuries** |  |  |  |  |
| Medial meniscus | 493 (36.1%) | 6 (22.2%) | 85 (30.9%) | 402 (37.8%) |
| Lateral meniscus | 326 (23.9%) | 7 (25.9%) | 64 (23.3%) | 255 (24.0%) |
| Cartilage injury | 474 (34.7%) | 2 (7.4%) | 75 (27.3%) | 397 (37.3%) |
| MCL | 9 (0.7%) | 0 | 1 (0.4%) | 8 (0.8%) |
| LCL | 2 (0.1%) | 0 | 0 | 2 (0.2%) |

ACL, anterior cruciate ligament; LCL, lateral collateral ligament; MCL, medial collateral ligament

**Table 5.** Demographic data of the study groups at the two-year follow-up

|  | **Total (n=1,211)** | **Paediatric (n=25)** | **Adolescent (n=234)** | **Young adult (n=952)** |
| --- | --- | --- | --- | --- |
| **Gender** |  |  |  |  |
| Male | 636 (52.5%) | 19 (76.0%) | 76 (32.5%) | 541 (56.8%) |
| Female | 575 (47.5%) | 6 (24.0%) | 158 (67.5%) | 411 (43.2%) |
| **Age at index surgery** | 24.9 (5.9) 25 (9; 35) | 13.7 (1.7) 15 (9; 15) | 17.2 (1.5) 17 (14; 19) | 27.0 (4.6) 27 (20; 35) |
| **Activity at ACL injury** |  |  |  |  |
| Pivoting sports | 705 (58.4%) | 13 (52.0%) | 160 (68.4%) | 532 (56.1%) |
| Non-pivoting sports | 33 (2.7%) | 1 (4.0%) | 6 (2.6%) | 26 (2.7%) |
| Martial arts | 36 (3.0%) | 1 (4.0%) | 5 (2.1%) | 30 (3.2%) |
| Winter sports | 175 (14.5%) | 4 (16.0%) | 28 (12.0%) | 143 (15.1%) |
| Other | 258 (21.4%) | 6 (24.0%) | 35 (15.0%) | 217 (22.9%) |
| Missing | 4 (0.3%) | 0 | 0 | 4 (0.4%) |
| **Groups of femoral fixation** |  |  |  |  |
| Cortical suspensory fixation | 517 (42.7%) | 16 (64.0%) | 95 (40.6%) | 406 (42.6%) |
| Adjustable cortical suspensory fixation | 167 (13.8%) | 6 (24.0%) | 31 (13.2%) | 130 (13.7%) |
| Screw fixation | 143 (11.8%) | 0 | 31 (13.2%) | 112 (11.8%) |
| Intratunnel transfixation | 375 (31.0%) | 3 (12.0%) | 76 (32.5%) | 296 (31.1%) |
| Other | 5 (0.4%) | 0 | 1 (0.4%) | 4 (0.4%) |
| Femur fixation missing | 4 (0.3%) | 0 | 0 | 4 (0.4%) |
| **Groups of tibial fixation** |  |  |  |  |
| Cortical suspensory fixation | 7 (0.6%) | 0 | 1 (0.4%) | 6 (0.6%) |
| Adjustable cortical suspensory fixation | 53 (4.4%) | 1 (4.0%) | 14 (6.0%) | 38 (4.0%) |
| Screw fixation | 868 (71.7%) | 19 (76.0%) | 165 (70.5%) | 684 (71.8%) |
| Bioabsorbable screw | 216 (17.8%) | 4 (16.0%) | 43 (18.4%) | 169 (17.8%) |
| Intratunnel transfixation | 36 (3.0%) | 0 | 8 (3.4%) | 28 (2.9%) |
| Other | 21 (1.7%) | 1 (4.0%) | 2 (0.9%) | 18 (1.9%) |
| Tibial fixation missing | 10 (0.8%) | 0 | 1 (0.4%) | 9 (0.9%) |
| **Concomitant injuries** |  |  |  |  |
| Medial meniscus | 433 (35.8%) | 7 (28.0%) | 80 (34.2%) | 346 (36.3%) |
| Lateral meniscus | 292 (24.1%) | 9 (36.0%) | 63 (26.9%) | 220 (23.1%) |
| Cartilage injury | 417 (34.4%) | 3 (12.0%) | 61 (26.1%) | 353 (37.1%) |
| MCL | 5 (0.4%) | 0 | 0 | 5 (0.5%) |
| LCL | 0 | 0 | 0 | 0 |

ACL, anterior cruciate ligament; LCL, lateral collateral ligament; MCL, medial collateral ligament

**Table 6.** Demographic data of the study groups at the five-year follow-up

|  | **Total (n=822)** | **Paediatric (n=9)** | **Adolescent (n=156)** | **Young adult (n=657)** |
| --- | --- | --- | --- | --- |
| **Gender** |  |  |  |  |
| Male | 424 (51.6%) | 4 (44.4%) | 54 (34.6%) | 366 (55.7%) |
| Female | 398 (48.4%) | 5 (55.6%) | 102 (65.4%) | 291 (44.3%) |
| **Age at index surgery** | 25.4 (5.9) 25 (9; 35) | 12.4 (2.1) 13 (9; 15) | 17.4 (1.5) 18 (14; 19) | 27.4 (4.6) 27 (20; 35) |
| **Activity at ACL injury** |  |  |  |  |
| Pivoting sports | 485 (59.3%) | 5 (55.6%) | 104 (66.7%) | 376 (57.6%) |
| Non-pivoting sports | 19 (2.3%) | 0 | 4 (2.6%) | 15 (2.3%) |
| Martial arts | 21 (2.6%) | 0 | 2 (1.3%) | 19 (2.9%) |
| Winter sports | 122 (14.9%) | 1 (11.1%) | 23 (14.7%) | 98 (15.0%) |
| Other | 171 (20.9%) | 3 (33.3%) | 23 (14.7%) | 145 (22.2%) |
| Missing | 4 (0.5%) | 0 | 0 | 4 (0.6%) |
| **Groups of femoral fixation** |  |  |  |  |
| Cortical suspensory fixation | 294 (35.8%) | 8 (88.9%) | 46 (29.5%) | 240 (36.5%) |
| Adjustable cortical suspensory fixation | 39 (4.7%) | 0 | 6 (3.8%) | 33 (5.0%) |
| Screw fixation | 125 (15.2%) | 0 | 28 (17.9%) | 97 (14.8%) |
| Intratunnel transfixation | 360 (43.8%) | 1 (11.1%) | 76 (48.7%) | 283 (43.1%) |
| Other | 1 (0.1%) | 0 | 0 | 1 (0.2%) |
| Femur fixation missing | 3 (0.4%) | 0 | 0 | 3 (0.5%) |
| **Groups of tibial fixation** |  |  |  |  |
| Cortical suspensory fixation | 5 (0.6%) | 0 | 0 | 5 (0.8%) |
| Adjustable cortical suspensory fixation | 4 (0.5%) | 0 | 0 | 4 (0.6%) |
| Screw fixation | 650 (79.1%) | 9 (100.0%) | 118 (75.6%) | 523 (79.6%) |
| Bioabsorbable screw | 104 (12.7%) | 0 | 25 (16.0%) | 79 (12.0%) |
| Intratunnel transfixation | 37 (4.5%) | 0 | 9 (5.8%) | 28 (4.3%) |
| Other | 15 (1.8%) | 0 | 3 (1.9%) | 12 (1.8%) |
| Tibial fixation missing | 7 (0.9%) | 0 | 1 (0.6%) | 6 (0.9%) |
| **Concomitant injuries** |  |  |  |  |
| Medial meniscus | 271 (33.0%) | 2 (22.2%) | 58 (37.2%) | 211 (32.1%) |
| Lateral meniscus | 172 (20.9%) | 2 (22.2%) | 43 (27.6%) | 127 (19.3%) |
| Cartilage injury | 276 (33.6%) | 1 (11.1%) | 45 (28.8%) | 230 (35.0%) |
| MCL | 2 (0.2%) | 0 | 1 (0.6%) | 1 (0.2%) |
| LCL | 0 | 0 | 0 | 0 |

ACL, anterior cruciate ligament; LCL, lateral collateral ligament; MCL, medial collateral ligament

**Table 7.** Demographic data of the study groups at the 10-year follow-up

|  | **Total (n=260)** | **Paediatric (n=3)** | **Adolescent (n=47)** | **Young adult (n=210)** |
| --- | --- | --- | --- | --- |
| **Gender** |  |  |  |  |
| Male | 121 (46.5%) | 0 | 12 (25.5%) | 109 (51.9%) |
| Female | 139 (53.5%) | 3 (100.0%) | 35 (74.5%) | 101 (48.1%) |
| **Age at index surgery** | 25.6 (6.0) 26 (11; 35) | 12.3 (1.2) 13 (11; 13) | 17.4 (1.5) 18 (14; 19) | 27.6 (4.7) 28 (20; 35) |
| **Activity at ACL injury** |  |  |  |  |
| Pivoting sports | 148 (57.8%) | 2 (66.7%) | 27 (57.4%) | 119 (57.8%) |
| Non-pivoting sports | 7 (2.7%) | 0 | 0 | 7 (3.4%) |
| Martial arts | 5 (2.0%) | 0 | 1 (2.1%) | 4 (1.9%) |
| Winter sports | 48 (18.8%) | 1 (33.3%) | 9 (19.1%) | 38 (18.4%) |
| Other | 48 (18.8%) | 0 | 10 (21.3%) | 38 (18.4%) |
| Missing | 4 (1.5%) | 0 | 0 | 4 (1.9%) |
| **Groups of femoral fixation** |  |  |  |  |
| Cortical suspensory fixation | 42 (16.2%) | 2 (66.7%) | 9 (19.1%) | 31 (14.8%) |
| Adjustable cortical suspensory fixation | 0 | 0 | 0 | 0 |
| Screw fixation | 45 (17.3%) | 0 | 8 (17.0%) | 37 (17.6%) |
| Intratunnel transfixation | 170 (65.4%) | 1 (33.3%) | 30 (63.8%) | 139 (66.2%) |
| Other | 3 (1.2%) | 0 | 0 | 3 (1.4%) |
| Femur fixation missing | 0 | 0 | 0 | 0 |
| **Groups of tibial fixation** |  |  |  |  |
| Cortical suspensory fixation | 0 | 0 | 0 | 0 |
| Adjustable cortical suspensory fixation | 0 | 0 | 0 | 0 |
| Screw fixation | 224 (86.2%) | 3 (100.0%) | 36 (76.6%) | 185 (88.1%) |
| Bioabsorbable screw | 4 (1.5%) | 0 | 1 (2.1%) | 3 (1.4%) |
| Intratunnel transfixation | 21 (8.1%) | 0 | 8 (17.0%) | 13 (6.2%) |
| Other | 8 (3.1%) | 0 | 1 (2.1%) | 7 (3.3%) |
| Tibial fixation missing | 3 (1.2%) | 0 | 1 (2.1%) | 2 (1.0%) |
| **Concomitant injuries** |  |  |  |  |
| Medial meniscus | 93 (35.8%) | 1 (33.3%) | 19 (40.4%) | 73 (34.8%) |
| Lateral meniscus | 47 (18.1%) | 0 | 10 (21.3%) | 37 (17.6%) |
| Cartilage injury | 95 (36.5%) | 0 | 8 (17.0%) | 87 (41.4%) |
| MCL | 3 (1.2%) | 0 | 1 (2.1%) | 2 (1.0%) |
| LCL | 1 (0.4%) | 0 | 0 | 1 (0.5%) |

ACL, anterior cruciate ligament; LCL, lateral collateral ligament; MCL, medial collateral ligament
